# Surgical Treatment of Pseudoaneurysm of the Ascending Aorta in a Patient with Sepsis

**DOI:** 10.21470/1678-9741-2020-0258

**Published:** 2021

**Authors:** Igor Zivkovic, Stefan Stankovic, Stasa Krasic, Miodrag Peric, Ivan Stojanovic

**Affiliations:** 1 Department of Cardiac Surgery, Dedinje Cardiovascular Institute, Belgrade, Serbia.; 2 Department of Cardiology, Mother and Child Health Institute of Serbia, Belgrade, Serbia.

**Keywords:** Aneurysm, False, Brachiocephalic Trunk, Aorta, Sepsis, Cardiovascular Diseases, Syncope

## Abstract

Pseudoaneurysm of the ascending aorta (PAA) is a hazardous and potentially fatal cardiovascular disease. This condition is caused by the rupture of at least one layer of the vessel and contained by the remaining vascular layers or the surrounding mediastinal structures. We presented the surgical treatment of a patient with sepsis and large PAA and brachiocephalic trunk, which was compressing the brachiocephalic trunk leading to syncope.

**Table:** 

Abbreviations, acronyms & symbols	
**CPB**	**= Cardiopulmonary bypass**
**MSCT**	**= Multi-slice computed tomography**
**PAA**	**= Pseudoaneurysm of the ascending aorta**
**RBC**	**= Red blood cells**
**WBC**	**= White blood cells**

## INTRODUCTION

Pseudoaneurysm of the ascending aorta (PAA) is a hazardous and potentially fatal condition. This disease is caused by the rupture of at least one layer of the vessel and contained by the remaining vascular layers or the surrounding mediastinal structures^[1]^. The incidence of PAA is less than 1%^[2]^. The etiology of PAA is related to previous cardiac surgery procedures, and the most common place of occurrence is at the aortic cannulation site. The proximal and distal aortic anastomoses are also common^[3]^. The location of PAA produces different clinical symptomatology; the most common symptoms are chest pain, pulsatile mass in the chest, compression of mediastinal structures, syncope^[4]^.

We describe the surgical treatment of a patient with sepsis and large a pseudoaneurysm of the aortic arch and brachiocephalic trunk.

## CASE PRESENTATION

### Clinical Data

A 62-year-old female patient, agitated, disorientated, with hypothyroidism and hypertension, was hospitalized due to a syncopal episode. The clinical examination was inadequate due to hemodynamic instability.

### Laboratory Analysis

Upon arrival, laboratory analyses showed WBC 35×109/L, hematocrit 25%, RBC 3,2×109/L, C-reactive protein level 418.9 mg/L, procalcitonin level 0.7 ng/mL, lactate dehydrogenase value was 746 U/L, and body temperature was 33°C, which led to a diagnosis of sepsis.

### Echocardiography

The echocardiography showed a 20-mm pericardial effusion and the color Doppler scan showed passable internal and external carotid arteries as well as vertebral arteries; however, left internal jugular and subclavian veins showed the presence of sizeable fresh coagulum. The aortic root was of regular dimension, and the ascending aorta was 39 mm, without sings of aortic dissection. To make the diagnosis, a multi-slice computed tomography (MSCT) was performed.

### Multi-Slice Computed Tomography (MSCT)

**Fig. 1:**
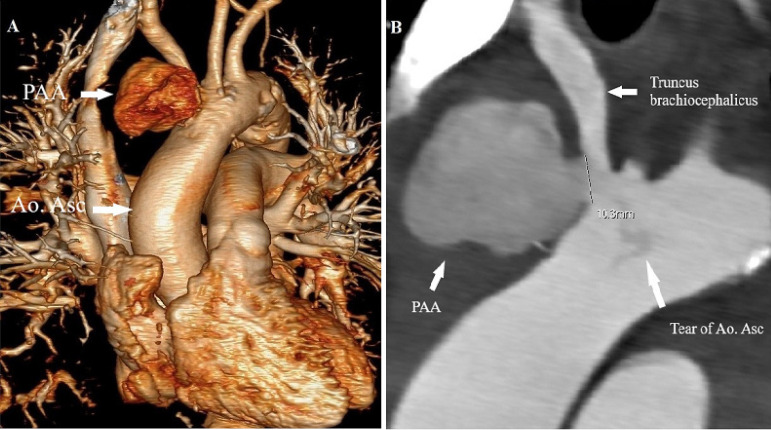
(A) MSCT volume rendering reconstruction showed a PAA near to the origin of the truncus brachiocephalicus. (B) MSCT scan reveals a 58 mm×58 mm PAA and truncus brachiocephalicus.

Computed tomography with contrast agent was performed and a 58×58 mm PAA with a 10 mm proximal neck, partially deriving from the aorta and partially from the brachiocephalic trunk, was discovered (Figure 1A and B). It contained a 22-mm circumferential coagulum, which was in sternal contact and compressing the brachiocephalic trunk. Due to the high risk of rupture of the pseudoaneurysm, it was decided that the patient undergoes surgery despite the septic condition.

## TECHNICAL DESCRIPTION

Cardiopulmonary bypass (CPB) was instituted by right femoral artery perfusion and right femoral venous drainage. After median sternotomy, the mediastinal swab was performed due to the presence of adhesions and suspicion of an inflammatory process.

**Fig. 2:**
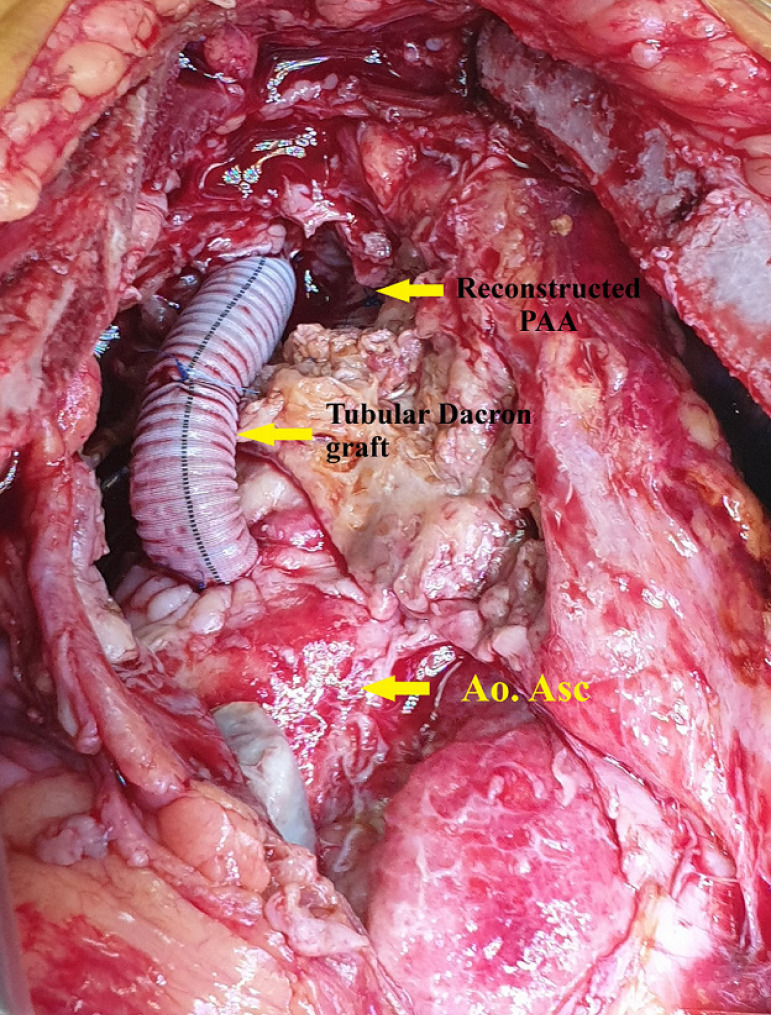
Surgical view of the reconstructed ascending aorta and truncus brachiocephalicus.

The patient's body temperature decreased to 25°C, and the aneurysm was incised during a hypothermic circulatory arrest. Surgical repair was performed with Dacron patch, which was placed and fixed in the part of the aortic arch where the brachiocephalic trunk separates from the aorta. Due to the poor quality of the brachiocephalic trunk tissue, debranching and reconstruction of the trunk with Dacron tubular were performed (Figure 2). De-airing maneuvers were performed, cardiopulmonary bypass was reestablished, and rewarming was carried out.

### Postoperative Outcome

**Fig. 3:**
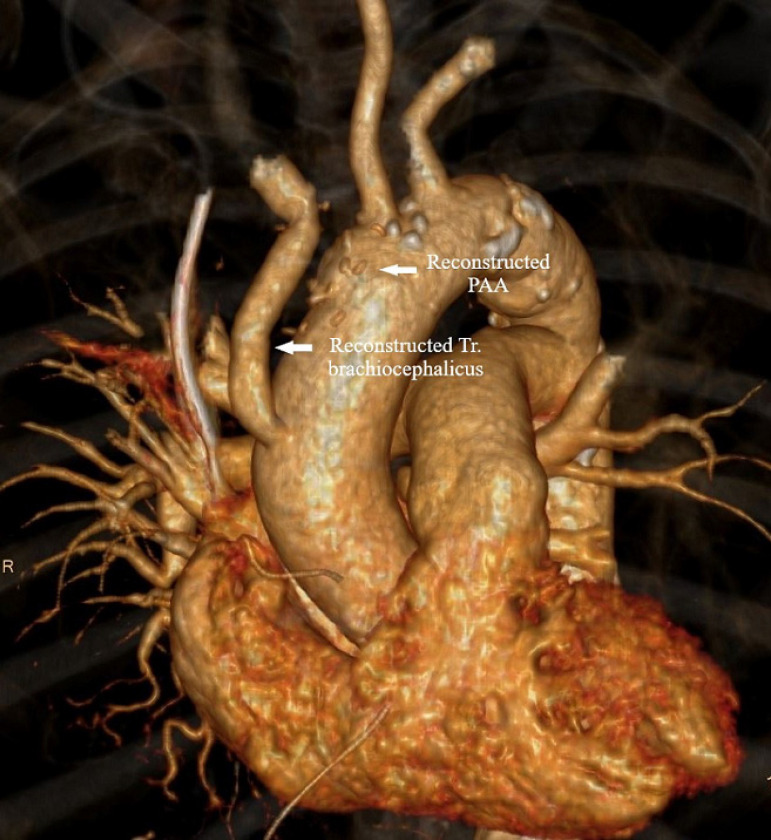
MSCT scan examination after surgical reconstruction of ascending aorta and truncus brachiocephalicus.

Postoperatively, there were no neurological or ischemic complications. The patient's blood analyses showed inflammatory factors decreasing; however, body temperature was back to normal. Hemoculture was obtained, and the patient was given empirical antibiotic therapy. After five days, hemoculture came back negative. Control computed tomography scan showed retrosternal air and fluid collection, as well as a 10-mm pericardial effusion with no signs of extravasation (Figure 3). The patient was discharged from the hospital on the 20^th^ postoperative day with no signs of pericardial effusion or extravasation, and laboratory results were within the normal range. The control echocardiography one month after hospital discharge was normal.

## DISCUSSION

PAA results from transmural rupture of the aortic wall, whit leakage contained by the surrounding structures^[2,5]^. The predisposing factors for PAA were the dissection of the native aorta, infection, connective tissue disorders, preoperative chronic hypertension, aortic calcification, infections, vasculitis, and rupture of the aortotomy site^[6]^. The incidence of PAA is less than 1%, and mortality in these patients is high^[2]^. Due to the high risk of rupture, a pseudoaneurysm requires an immediate operation when diagnosed. Untreated patients had a high mortality rate of 29 to 46% due to fatal bleeding^[7]^. About 20% of patients with PAA had sepsis-related symptoms^[5]^. Our case had a similar clinical picture.

Despite recent reports of percutaneous exclusion of false aneurysms, surgery is still necessary in most cases and has presented definitive treatment options^[4]^.

PAA surgery is seldomly performed without technical challenges, even more in this specific case, considering that the patient was in a septic state. Even though there are different variations of establishing CPB when performing this type of surgery, femoro-femoral bypass and deep hypothermia have been widely used with satisfactory results^[7]^. This strategy prevented PAA rupture during sternotomy and tissue preparation with following exsanguination^[2,5]^.

Aortic arch pseudoaneurysm is a life-threatening condition that requires immediate surgery. Even though the patient was in a septic state and the mortality rate is high, although there is a high risk of operative treatment, surgery could be performed with satisfactory results.

**Table:** 

Authors' roles & responsibilities	
IZ	Substantial contributions to the conception or design of the work; or the acquisition, analysis, or interpretation of data for the work; final approval of the version to be published
SS	Substantial contributions to the conception or design of the work; or the acquisition, analysis, or interpretation of data for the work; final approval of the version to be published
SK	Drafting the work or revising it critically for important intellectual content; final approval of the version to be published
MP	Final approval of the version to be published
IS	Agreement to be accountable for all aspects of the work in ensuring that questions related to the accuracy or integrity of any part of the work are appropriately investigated and resolved; final approval of the version to be published
